# Outcome prediction with resting-state functional connectivity after cardiac arrest

**DOI:** 10.1038/s41598-020-68683-y

**Published:** 2020-07-16

**Authors:** Franca Wagner, Matthias Hänggi, Anja Weck, Manuela Pastore-Wapp, Roland Wiest, Claus Kiefer

**Affiliations:** 1 Department of Diagnostic and Interventional Neuroradiology, Support Center for Advanced Neuroimaging (SCAN), Inselspital, Bern University Hospital, University of Bern, Freiburgstrasse 10, 3010 Bern, Switzerland; 2 Department of Intensive Care Medicine, Inselspital, Bern University Hospital, University of Bern, Bern, Switzerland

**Keywords:** Neuroscience, Biomarkers, Health care, Neurology, Mathematics and computing

## Abstract

Predicting outcome in comatose patients after successful cardiopulmonary resuscitation is challenging. Our primary aim was to assess the potential contribution of resting-state-functional magnetic resonance imaging (RS-fMRI) in predicting neurological outcome. RS-fMRI was used to evaluate functional and effective connectivity within the default mode network in a cohort of 90 comatose patients and their impact on functional neurological outcome after 3 months. The RS-fMRI processing protocol comprises the evaluation of functional and effective connectivity within the default mode network. Seed-to-voxel and ROI-to-ROI feature analysis was performed as starting point for a supervised machine-learning approach. Classification of the Cerebral Performance Category (CPC) 1–3 (good to acceptable outcome) versus CPC 4–5 (adverse outcome) achieved a positive predictive value of 91.7%, sensitivity of 90.2%, and accuracy of 87.8%. A direct link to the level of consciousness and outcome after 3 months was identified for measures of segregation in the precuneus, in medial and right frontal regions. Thalamic connectivity appeared significantly reduced in patients without conscious response. Decreased within-network connectivity in the default mode network and within cortico-thalamic circuits correlated with clinical outcome after 3 months. Our results indicate a potential role of these markers for decision-making in comatose patients early after cardiac arrest.

## Introduction

Disorders of consciousness are a consequence of pathologies that affect an individual’s ability to interact with the external world. Many factors contribute to disruption of consciousness, e.g., metabolic, epileptic, traumatic, and hypoxemic events, eventually ending in severe brain damage^1^. Disruption of consciousness is categorized by different levels of quantitative impairment: to describe global states as levels of consciousness implies that there are degrees of consciousness and that changes in the global state of consciousness can be represented as changes along a clinical scoring system. Three distinct “stages” of altered consciousness have been described—coma, vegetative state (VS), and minimally conscious state. The differentiation between the stages is based on behavioral criteria^2^. Many coma scales have been developed; these include the Glasgow Coma Scale (GCS), Grady Coma Scale, Comprehensive Level of Consciousness Scale, Full Outline of UnResponsiveness Score, Disorders of Consciousness Scale, Disability Rating Scale, Innsbruck Coma Scale, and the Glasgow Liege Coma Scale^3^. However, “grading a degraded level” of consciousness; and consequently outcome prediction in comatose patients may be particularly challenging.

A better understanding of the neural correlates underlying altered states of consciousness can be gained from functional neuroimaging investigations, e.g., resting-state functional magnetic resonance imaging (RS-fMRI) and analyses of regional connectivity^4^ RS-fMRI focuses on spontaneous, low-frequency fluctuations (< 0.1 Hz) in the blood oxygen level-dependent (BOLD) signal. The functional significance of these fluctuations was first noted by Biswal in 1995^5^. Fundamental resting-state networks (RSN) encompass the default mode network (DMN), including the precuneus/posterior cingulate cortex (PCC), mesiofrontal/anterior cingulate cortex (ACC), and temporoparietal junction areas and the hippocampi^6^. The DMN is a high-level cognitive RSN, initially defined as the regions that are more active at rest than in any goal-oriented cognitive activity^7^. Its putative involvement in self-awareness makes it an attractive network in which to assess the disorders and levels of consciousness. The DMN was first identified from positron-emission tomography (PET) data by Raichle et al*.*^7^ and Greicius et al*.* using fMRI^8^ and confirmed in many studies using a variety of methods^9–15^. External awareness correlates with BOLD fluctuations within the frontoparietal network (FPN), also known as the task-positive network. The latter encompasses mainly lateral frontoparietal hemispheric regions^16^ reflecting the brain activations during goal-directed behavior and it has been linked to cognitive processes of external sensory input, such as somatosensory^17^ visual^18^ and auditory^19^. Integrity of cortico-cortical and thalamo-cortical connectivity impacts on the behavioral profiles, and the complex information integration sustaining conscious awareness, have been investigated in both structural and neurophysiological studies^20–22^. Dynamic patterns of information coded within spatially distributed but anatomically connected neural networks originate from the thalamo-cortical circuits^23^ and connect them with cortical loops by reciprocal connections. Yao et al*.*^24^ demonstrated attenuated thalamic activation in patients with diffuse axonal brain injury, and a negative correlation with the GCS score, showing that the thalamus is crucial for maintaining consciousness^25^.

The presence of functional DMN connectivity in patients with reversible coma is supported by previous studies and case reports across a range of altered states of consciousness including light^30^ and deep^31^ anesthesia, minimal consciousness^32^ VS^32–35^, and coma^32,36^. The finding of altered connectivity in the DMN along various states of altered consciousness suggests that the DMN represents a fundamental or intrinsic property of functional brain organization^31^.

PET studies revealed that the precuneus underwent the largest recovery-related metabolic changes during the recovery of consciousness after VS^37^. The posterior cingulate cortex/precuneus areas encompassed by the DMN have been shown to possess both structural and functional hub properties in humans^38–40^ and seem to be important for integration of multiple cognitive processes, including consciousness.

The primary aim of our study was to predict outcome in a cohort of comatose patients after successful cardiopulmonary resuscitation (CPR) in order to assess the potential contribution of RS-fMRI in the early prognostication of neurological outcome. We investigated the DMN functional connectivity and the thalamo-cortical circuit, especially prefrontal-thalamic connectivity in the study patients with an indeterminate prognosis. We hypothesized that the degree of those relationships would correlate with the functional outcome of those patients after 3 months.

Furthermore, we proposed that the severity of coma is related to the level of functional connectivity in the networks responsible for conscious processing and as such predicts outcome in comatose patients with irreversible hypoxic-ischemic brain damage. Unfavorable neurological outcome would be correlated to absent/altered DMN connectivity and thalamo-cortical directed connectivity, whereas comatose patients with reversible coma and a good functional outcome would have intact DMN and thalamo-cortical connectivity.

## Materials and methods

This study was performed in a prospective fashion with blinded endpoint (clinical outcome) assessment. The Bern Cantonal Ethic Committee (Gesundheits-, Sozial- und Integrationsdirektion des Kantons Bern, Kantonale Ethikkommission für die Forschung, 3010 Bern, Switzerland; Nr 116/15, 25/NOV/2015) approved this study, and all research was performed in accordance with the Swiss Federal Act on Research involving Human Beings (Human Research Act, HRA) and the Declaration of Helsinki. Since MRI is performed routinely in comatose survivors after CPR in the intensive care unit (ICU) in Bern, informed consent for the additional fMRI sequences was waived for those patients who never regained consciousness and did not survive hospital admission. For surviving patients, the informed consent was deferred until follow-up and obtained by the patient, or next of kin or legal representative, depending on consciousness.

This prospective, exploratory study was performed in a multidisciplinary ICU (Department of Intensive Care Medicine, Inselspital, University of Bern) with 37 beds. All patients admitted to the ICU following CPR after circulatory arrest between January 2016 and March 2019 were eligible.

### Patients

**Figure 1:**
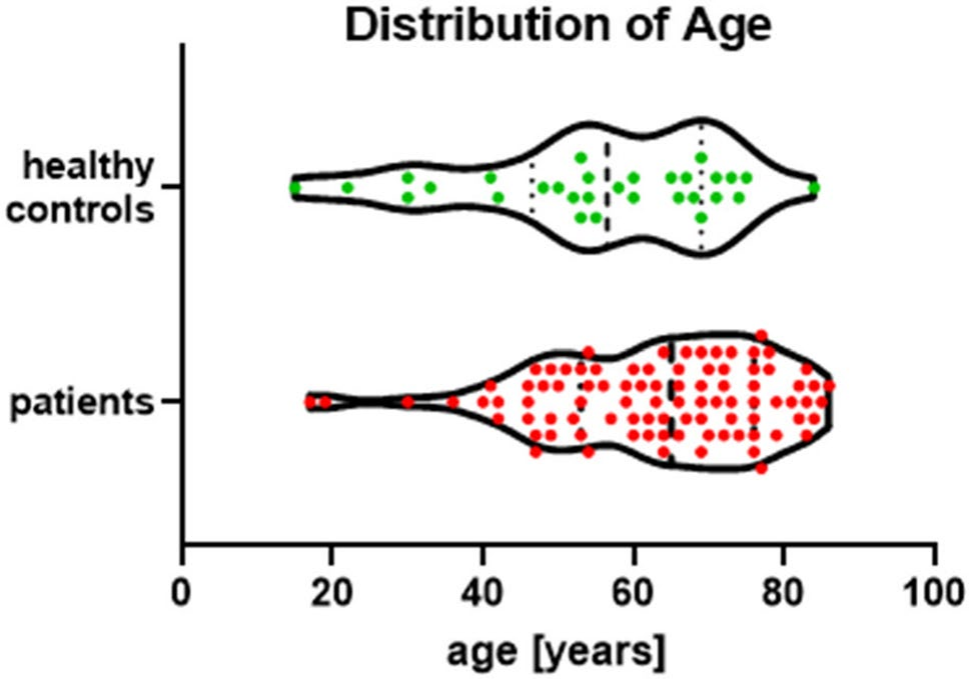
Age distribution of all patients in comparison to the healthy control group.

Patients were eligible for inclusion in the study if the following criteria were met: out-of-hospital cardiac arrest of presumed cardiac origin, age ≥ 18 years, persistent coma with GCS ≤ 7, partially or completely preserved brainstem reflexes in clinical exam, and the possibility to perform MRI within 48 h after cardiac arrest. The mean age of the 30 patients in the CPC 1–3 subgroup was 58.4 years with a range from 20 to 83 years. For the patients in the CPC 4–5 subgroup, the mean age was 63.6 years with a range from 41 to 86 years. All participants in the healthy control groups were ≥ 18 years old. Their mean age was 55.4 years, with a range from 18 to 84 years. The age distribution of all patients in comparison to the healthy control group is shown in Fig. 1. Exclusion criteria were: previous structural brain lesions such as stroke, neurodegenerative disorders, tumor, or coma due to intoxication, hypoglycemia, drowning, hanging or strangulation, and advance directives to limit ICU treatment.

All study variables were obtained from the designated resuscitation register. Recorded variables included general demographic data, etiology of circulatory arrest, co-morbidities, total duration of CPR and time from start of CPR to return of spontaneous circulation (“down time”), GCS and cerebral performance category (CPC) on admission to the ICU, GCS and CPC while in the ICU, CPC after 3 months in survivors, and results of clinical neurological examination including brainstem reflexes and electroencephalography (EEG).

The clinical outcome measure (reference standard) was neurological status expressed as the best score at the time of discharge from the ICU and within 3 months after CPR, according to the Pittsburgh CPC^41^. CPC is a widely used assessment of the functional status of cardiac arrest patients. CPC scores were determined by a telephone interview with either the patient or his/her legal representative, or from a review of the patient’s charts during a subsequent hospital admission. CPC = 1 indicates no or minor neurological deficits, CPC = 2 moderate disability, CPC = 3 severe disability, CPC = 4 unresponsive wakefulness state and CPC = 5 death. Although patients with CPC = 3 may show further recovery, we classified CPC = 1–3 as good/favorable outcome and CPC = 4–5 as poor/unfavorable outcome, according to Grippo et al*.*^42^.

All participants of the healthy control group all gave their written consent for the MR scan for the study.

### General care of the patients

All patients received full intensive care for at least 2 days after CPR. Sedation was kept to a minimal level to ensure adequate mechanical ventilation. Hemodynamic management was performed according to a standard protocol^43^. Targeted temperature management (TTM) at 33–36 °C was applied using feedback devices (Alsius/Arctic Sun) followed by slow rewarming^44^. Blood glucose management aimed at a range of 4.5–7.0 μmol/L.

In patients with persistent coma after rewarming, prognosis was assessed by the physician in charge based on clinical examination, EEG results, and structural MRI, supplemented by neuron-specific enolase and somatosensory evoked potential. The results of the advanced neuroimaging procedures (RS-fMRI) were not used in clinical decision-making. If the prognosis was considered potentially moderate or good, full treatment was continued, including tracheotomy when necessary. If prognosis was considered poor, according to the criteria commonly used and specified in the TTM trial, the outlook was discussed with the patient’s family taking into account instructions in the patient's will or in accordance with an existing patient directive. Since this was a trial within a clinical scenario, no formal withdrawal of life support protocol was followed, but all results reported here are from patients who had positive results for at least two prognostic markers of unfavorable outcome. When agreement was reached, active treatment was stopped; the patient was extubated and palliative care was continued until the patient died.

### Magnetic resonance imaging

Image acquisition was performed with one of our institutional 3 T Siemens MR Scanners (Magnetom Vida, Magnetom Verio or Magnetom Skyra_fit; Erlangen, Germany). The imaging protocol on the 3 T scanners was the same for all patients. This protocol included diffusion-weighted imaging with calculation of the apparent diffusion coefficient, axial T2w, coronal T2w-FLAIR, T1w multiplanar-reconstruction sequence (MPR-T1), 3D time-of-flight angiography, susceptibility-weighted imaging, contrast-enhanced T1w turbo spin echo and contrast-enhanced T1w multiplanar-reconstruction sequence (MPR-T1).

The fMRI sequence was a simultaneous multi-slice with echo-planar imaging (SMS-EPI), TR/TE = 300/30 ms, N = 32 slices, multiband acceleration factor 8, matrix 64, voxel 3.6 mm iso, M = 1,000 measurements, flip-angle 30° (short TR-related Ernst angle for gray matter at 3 T).

### Resting-state functional MRI

MRI-based determination of functional connectivity benefits from parallel imaging using simultaneous multi-slice EPI^45^. Machine-learning algorithms^46,47^ and powerful computer hardware can be employed to identify the disruptions of connectivity that eventually lead to reduced cognitive function. Random forest classifiers is a method of machine learning that combines different decision trees within a flowchart-like structure, in which each internal node represents a "test" on an attribute, into a single model.

To make a prediction, each decision tree in the forest considers a random subset of features when forming questions having access to a random set of the training data points. This increases diversity in the forest leading to more robust overall predictions. For classification purposes encompassing discrete class labels such as prognostic outcome markers, the random forest will take a majority vote for the predicted class. Breiman^47^ first proposed random forests, which add an additional layer of randomness to bagging^46^. In addition to creating each tree using a different bootstrap example of the data, random forests change the way the classification or regression trees are created.

In standard trees, each node is split using the best split among all variables. In a random forest, each node is split using the best among a subset of predictors randomly chosen at that node. This somewhat counterintuitive strategy outperforms many other classifiers, including discriminant analysis, support vector machines, and neural networks and is robust against overfitting^47^. Random forests are computationally effective, offer good prediction performance, and show very low sensitivity to noise. The algorithm only needs two parameters, the number of variables in the random subset at each node and the number of trees in the forest. Moreover, there is no need for cross-validation or a separate test set to get an unbiased estimate of the classification error and misclassification rate and the variable importance (e.g. Gini score)—it is estimated internally during the run as the training sets of individual trees are constructed by bootstrap replication. On average, there is 1/e ~ 36.8% of instances not taking part in construction of the tree. These “out-of-bag” instances are the source of data for useful internal estimates of error, strength, and correlation.

In order to analyze the Pearson-related resting-state functional connectivity, the Matlab (R2014a)-based CONN (v13.1)-toolbox (https://web.conn-toolbox.org/) was used^48^. It offers characterization of functional brain connectivity for both normal and patient populations.

1. The CONN analysis of the SMS-EPI data and the anatomical 3D-T1 MPRAGE dataset comprised preprocessing, where possible confounds in the BOLD signal were defined, explored, and removed by suitable segmentation, slice-timing, realignment, co-registration, normalization, and smoothing steps. This was followed by a first-level analysis, where seeds of interest were defined to explore the functional connectivity of different sources separately for each subject based on a series of predefined regions of interest and template atlases.
2. Functional connectivity values from different sources at single-subject level, seed-to-voxel, and ROI-to-ROI mode were computed, and related connectivity maps for each selected source (as well as complete ROI-to-ROI connectivity matrices for these sources) for each subject and for each condition were stored for further processing steps outside the CONN-framework.
3. The data matrix for further statistical analysis outside CONN encompasses the subjects' correlation matrices (Fisher-transformed z-scores) of the raw connectivity values. The correlation coefficients are converted to normally distributed scores using Fisher’s transformation (an inverse hyperbolic tangent function is applied to all bivariate and semipartial correlation measures to improve the normality assumptions of standard second-level general linear models) (see Fig. 2).

**Figure 2:**
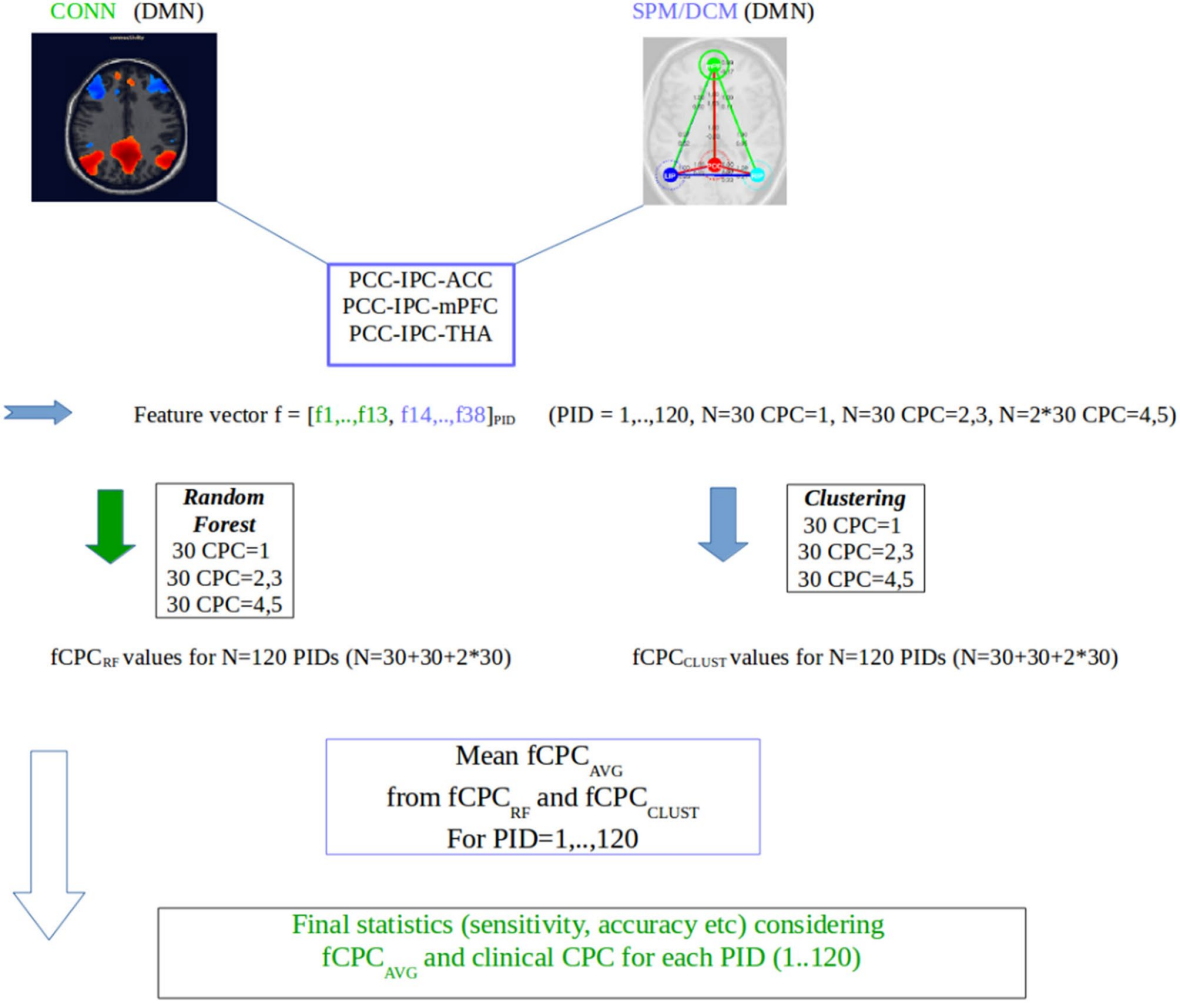
RS-fMRI evaluation strategy.
4. To avoid statistical bias due to focusing on one classification method or certain software packages we extended the computation of the classification scores by an additional optimized fuzzy-c means technique, known as Gustafson–Kessel (GK)^49^ and the effective connectivity dynamic causal modeling (DCM) approach of Friston et al*.*^50^, used in SPM12. Within the effective connectivity DCM framework, the coupling (A) values were used to construct and extend the feature vector. DCM analysis was based on our a-priori hypothesis that cortico-cortical and cortico-thalamic connectivity within the core structures of the DMN is reduced or absent in comatose patients. We restricted our analysis to the PCC-IPC-ACC, PCC-IPC-mPFC, PCC-IPC-THA (precuneus, inferior parietal lobe left/right, anterior cingulate cortex left/right, medial prefrontal cortex, thalamus (Tda,Tdb,Tde,Tdf)), and the components 14–38 from DCM (ACC, mPFC, THA) using the CSD-analysis option (SPM12), considering the first-level covariates such as realignment/movement from the CONN analysis parameters^48,50^. The classification-relevant feature components were computed from the A-probability-weighted directional connectivity for the respective networks (conditional expectation and probability values). The resulting feature vector used for clustering and random forest analysis is a 38-dim vector comprising the z-score components 1–13 from CONN and the components 14–38 from DCM. Based on this 38-dim vector, every patient was assigned to one of the two defined groups CPC 1–3 or CPC 4–5.
5. Random forest classification (implemented in Matlab R2014a): the number of trees was set to default (= 500). The classification was performed with respect to CPC 1–3 versus CPC 4–5 and the mean classifiers derived from the random forest and GK-cluster analysis (Fig. 2). The variables (feature components) with Gini score values > 0.4 were considered important for classification.

## Results

### Patients

Between January 2016 and March 2019, 90 patients underwent MRI of the brain with RS-MRI after CPR. They were included in this study and compared to 30 healthy controls. The demographic characteristics of the 90 patients and 30 healthy controls are summarized in Table 1. There were no significant differences in age between the healthy control group and the survivors (t = 1.01; p = 0.32) but the age of the non-survivors compared to controls was significantly higher (t = 2.85; p = 0.005). Between the 30 survivors and the 60 non-survivors, there was no difference in age (t = 1.51, p = 0.135) or sex (χ^2^ = 0.28, p = 0.60). Mean time to MRI was 48.5 h for survivors and 45.8 h for non-survivors and differed not significant.

**Table 1:** Patient demographics.

	CPC 1–3 (n = 30)	CPC 4–5 (n = 60)	Healthy controls (n = 30)
Age (years; mean)	58.4 (range 20–83)	63.6 (range 41–86)	55.4 (range 18–84)
Sex (male:female)	23:7	47:13	13:17
Time to MRI (hours) range	48.5 27–112	45.8 11–96	Elective scans
CPC at ICU discharge	1 = 0 2 = 11 3 = 19	4 = 4 5 = 56	1 = 30
CPC after 3 months	1 = 13 2 = 16 3 = 1	4 = 0 5 = 60	1 = 30

*CPC* cerebral performance category, *ICU* intensive care unit, *MRI* magnetic resonance imaging.

### Resting-state functional MRI

The misclassification rate in this two-class problem was 25%, which implies that correlations between the variables under investigation impact on the classification and discrimination. The statistical results for correct classification (power) of CPC 1–3 versus CPC 4–5 were as follows: positive predictive value: 91.6667%, sensitivity: 90.1639%, accuracy: 87.7778%, specificity: 82.7586%, negative predictive value: 80%, false-positive rate: 17.2414%, false-negative rate: 9.8361%, false discovery rate: 8.3333%, false omission rate: 20%. The dominant feature components according to the Gini score (f1–13: CONN, f14–38: DCM) were PCC > IPC r (1), PCC > IPC l (2), PCC > ACC r (4), PCC > THA (8, Tdb251), PCC > mPFC (13), PCC > ACC (15), ACC > IPC l (20), IPC l > IPC l (24), IPC r > IPC r (29) ('>' indicates the directed flow of information; the THA-ROI Tdb 251 is the connection between right thalamus and brainstem). In some cases (dominance of the ACC network) the PCC > THA (Tda132) and the IPC > ACC feature components were also significant in the random forest sense (Gini score).

## Discussion

In this study, we used a supervised machine-learning approach based on a combination of functional and effective connectivity matrices within the DMN to determine the severity of coma and predict clinical outcome in a prospective cohort of comatose patients following cardiac arrest and resuscitation. Neurological outcome after resuscitation correlated well with the level of disrupted connectivity within the DMN and could be determined by subsequent random forest classification and fuzzy clustering. Our statistical results for correct classification of CPC 1–3 (good/favorable neurological outcome) versus CPC 4–5 (poor/unfavorable neurological outcome) confirmed disrupted connectivity in all non-survivors, with an excellent yield for classification between CPC 1–3 and CPC 4–5.

Our data further suggest that the level of functional and effective connectivity between involved nodes of the DCM could represent an early biomarker to effectively distinguish between survivors and non-survivors.

Our results support the findings of Norton et al*.*^51^ who studied 13 patients 1–6 days after cardiac arrest. The authors found that DMN activation and connectivity was preserved in the two patients who had regained consciousness at the 3-month reevaluation, whereas DMN activity could not be identified by the initial fMRI scanning in the 11 patients who remained unconscious after 3 months.

In a study of 17 patients who underwent RS-fMRI 4–7 days after cardiac resuscitation, Koenig et al*.*^52^ reported decreased DMN connectivity compared with healthy controls and an association between CPC at discharge and precuneus and PCC connectivity. In a separate study of patients with trauma and anoxic coma (14 and 13 patients, respectively), PCC connectivity with brain areas that are normally synchronized with the PCC was significantly disrupted^53^. These findings were further strengthened by a meta-analysis of resting-state neuroimaging studies of patients with disorders of consciousness, which demonstrated significantly reduced activity in nodes of the DMN^54^.

The results of the present study add to the existing body of evidence from the literature showing that connectivity within the DMN and thalamo-cortical projection pathways is crucial for the maintenance of consciousness after a brain insult.

Functional MRI constitutes a complementary diagnostic tool during early-stage (< day 3) coma to support clinical decisions after successful cardiac resuscitation. Our results demonstrate that altered connectivity within key nodes of the DMN is observed within 48 h after the onset of hypoxic brain injury following cardiac arrest and resuscitation, and correlates with the patient’s long-term neurological outcome.

The connectivity strength within fronto-parietal and thalamo-cortical loops differed between patients and controls. Reduced connectivity between the precuneus and IPS, ACC, and thalamus revealed differences in the efficiency of information transfer in patients with CPC 1–3 vs. CPC 4–5. Compared to the healthy controls, all patients demonstrated decreased seed-to-voxel connectivity, reduced embeddedness of the PCC, and reduced local efficiency of the precuneus. In medial frontal regions like the ACC, the interaction with the IPC was decreased.

Our findings indicate alterations in the ROI-based centrality, especially in the medial frontal cortex. A shift of increased functional connectivity toward the left frontal regions was noted, particularly for patients with minimally conscious responses. Most of the affected regions in unconscious patients who had a negative neurological outcome (CPC 4 or CPC 5) after CPR were part of a set of highly central nodes that are more densely interconnected, such as medial parietal regions, the superior frontal gyrus, the ACC, and the thalamus^55^. These nodes act as a strongly interlinked entity and are suggested to play a central role in overall brain communication, with connections tending to span long distances enabling highly efficient integration. Thus, impairments of the functional connectivity of these regions reflect dysfunctions in overall information integration and have an impact on many different cognitive functions. Importantly, there is some overlap between regions of the rich club and the DMN^8,56^, which is altered when consciousness is impaired. According to theories of neural correlation of consciousness, the thalamus plays a key role^23,57,58^. It is highly reciprocally connected with cortical areas, especially with frontal regions, and is also a region belonging to the rich club nodes.

Our hypothesis that imaging correlates of consciousness are reflected by a dynamic pattern of information binding within spatially distributed but highly connected neural networks, centered along thalamo-cortical circuits, emphasizes the critical role of thalamic connectivity^23^ and is confirmed by our study. Previous studies investigating impaired consciousness provided evidence for a crucial role of the thalamus^25–29^. However, most of these studies did not compare unconscious patients with different stages of consciousness and their neurological outcomes directly. Moreover, they were conducted with relatively small samples of patients. In contrast, our study examined the data of 90 patients after CPR with different stages of unconsciousness after cardiac arrest at the time of the cardiac event and at the 3-month follow-up assessment, and compared them to 30 healthy controls.

A novel finding of this study is that the precuneus differed significantly between the two patient groups in its efficiency of local information transfer. This is in line with a previous investigation whose findings indicated that medial parietal regions are particularly sensitive to differences in functional connectivity^32^. The efficient embeddedness of this area seems to hold a key position in impaired consciousness. It is sensitive to different levels of consciousness and could be useful in outcome prediction for patients after cardiac resuscitation. This finding supports the postulation of Laureys and Schiff^59^ that the medial parietal regions are closely associated with recovery of consciousness. The results concerning functional connectivity complement the picture, showing reduced connectivity between almost all regions of the FPN in all patients with reduced connectivity in the CPC 4–5 patient group compared to the CPC 1–3 group. Interestingly, analysis of segregation differentiated between good/favorable (CPC 1–3) and poor/unfavorable (CPC 4–5) patients. One explanation for this finding may be that the integration of thalamic regions is not required to maintain lower levels of consciousness but is needed for intact conscious awareness and content. In contrast, segregation of the main regions within the FPN seems to play a primary role in recovery of consciousness according to the study of Crone et al*.*^60^. Further studies are warranted to gain a better insight into the role of PCC–thalamic interactions in conscious awareness, which suggest an alternative anatomical substrate of consciousness to the FPN^61^.

The relevance of RS-fMRI needs to be confirmed in prospective multicenter studies to define clear prognostic algorithms for comatose patients after cardiac arrest at an early stage and to disentangle the mechanisms of interaction between distinct regions of the brain to understand impairment of consciousness in patients after cardiac arrest. Further, data should be compared to the gold standard of current prognostication according to established international recommendations.

This study has some limitations: MRI scans were performed between 27 and 112 h (mean 48.5 h) after cardiac arrest in the survivors and between 11 and 96 h (mean 45.8 h) after cardiac arrest in the non-survivors, which confounds the between-group comparison of the classification. However, this reflects clinical practice. For further studies, strict timing of MRI with regard to onset of cardiac arrest is desirable. Second, all patients required sedation before RS-fMRI. Although deep sedation has been shown to reduce DMN connectivity, the dose of sedatives would not be expected to cause the observed degree of DMN disruption^62,63^. Investigations in nonhuman primates have shown that the effect of sedation on connectivity was minimal when the DMN was observed under deep anesthesia^31^.

Additionally, the impact of age-related alterations in brain connectivity is a topic of ongoing research, and some conflicting results have been published in the literature. While several studies report decreased functional connectivity in elderly subjects, region-wise increases have also been reported^64^. The majority of these studies compared only healthy subjects^65–67^, while some studies focused on different age-groups in the elderly population. Siman-Tov et al.^68^ investigated functional connectivity in different age groups (young, middle-aged, and old) and reported connectivity alterations starting in the middle-aged group, suggesting that aging-related neural changes are present during early adulthood. Farràs-Permanyer et. al.^69^ reported non-linear functional connectivity alterations in different age groups, with improvements in the oldest subjects (≥ 80 years old). All previous studies investigating age-related functional connectivity have been conducted in healthy subjects. Since data on age-related connectivity modulation are non-in conjunction with prognostication of outcome in comatose patients after resuscitation, this issue was outside the scope of our current study. Since only a few of our patients were younger than 45 years, valid conclusions cannot be drawn from the current dataset.

A further weakness of this study is the lack of protocolled withdrawal of life support. This puts the patients and the study results at risk of the so-called self-fulfilling prophecy. To minimize this risk, we did not include patients whose withdrawal of life support was based on opinion and/or on presumed wishes of the family. Limiting life support and assisted suicide have been legal in Switzerland for more than 50 years, so the culture of forgoing active treatment if quality of life cannot be assured is widespread. A possible future trial should include an appropriate protocol. On the other hand, our results demonstrate a relatively high predictive value within 48 h after cardiac arrest. Since almost all examinations predict poor outcome with a relatively high certainty, only reactivity to external stimulus during EEG recording has the ability to predict awakening. If these results can be confirmed, fMRI can add diagnostic accuracy to predict awakening and unfavorable outcome soon after CPR. An advantage of our study is that it was register-based the selection bias may therefore be reduced.

## Conclusion

Our results indicate the diagnostic and prognostic value of early RS-fMRI in comatose patients after cardiac arrest and successful resuscitation. Correlation analysis of neurological outcome from ROI-based RS-fMRI within the DMN network disruption outperformed merely clinical classification methods, notably for identifying patients who recovered to baseline. RS-fMRI may be considered a potential additional marker in the complex process of decision-making for comatose patients in the early period after cardiac arrest. Automated image processing may contribute to the optimization of early neurological outcome prediction and further therapeutic management of these patients.
